# Evaluating the impact of enhanced recovery after surgery protocols following oesophagectomy: a systematic review and meta-analysis of randomised clinical trials

**DOI:** 10.1093/dote/doae118

**Published:** 2025-01-10

**Authors:** Patrick Kennelly, Matthew G Davey, Diana Griniouk, Gavin Calpin, Noel E Donlon

**Affiliations:** Department of Surgery, Royal College of Surgeons in Ireland (RCSI), Dublin, Ireland; Department of Surgery, Royal College of Surgeons in Ireland (RCSI), Dublin, Ireland; Department of Surgery, Royal College of Surgeons in Ireland (RCSI), Dublin, Ireland; Department of Surgery, Royal College of Surgeons in Ireland (RCSI), Dublin, Ireland; Department of Surgery, Royal College of Surgeons in Ireland (RCSI), Dublin, Ireland

**Keywords:** enhanced recovery after surgery, ERAS, esophagectomy, oesophagectomy, patient outcomes

## Abstract

Enhanced Recovery After Surgery (ERAS) protocols are evidence-based care improvement pathways which are perceived to expedite patient recovery following surgery. Their utility in the setting of oesophagectomy remains unclear. The aim of this study was to perform a systematic review and meta-analysis of randomised clinical trials (RCTs) to evaluate the impact of ERAS protocols on recovery following oesophagectomy compared to standard care. A systematic review was performed in accordance with preferred reporting items for systematic reviews and meta-analyses guidelines. Meta-analysis was performed using Review Manager (Version 5.4). Six RCTs including 850 patients were included in this meta-analysis. Overall complication rate (Odds Ratio (OR): 0.35, Confidence Interval (CI): 0.21, 0.59, *P* < 0.0001), pulmonary complications (OR: 0.40, CI: 0.24, 0.67, *P* = 0.0005), post-operative length of stay (LOS) (OR -1.88, CI -2.05, −1.70, *P* < 0.00001) and time to post-operative flatus (OR: -5.20, CI: −9.46, −0.95, *P* = 0.02) favoured the ERAS group. There was no difference noted for anastomotic leak (OR: 0.55, CI: 0.24, 1.28, *P* = 0.17), cardiac complications (OR: 0.86, CI: 0.30, 2.46, *P* = 0.78), gastrointestinal complications (OR: 0.51, CI: 0.23, 1.17, *P* = 0.11), wound complications (OR: 0.85, CI: 0.28, 2.58, *P* = 0.78), mortality (OR: 1.37, CI: 0.26, 7.4, *P* = 0.71), and 30-day re-admission rate (OR: 1.29, CI: 0.30, 5.47, *P* = 0.73) between ERAS and standard care groups. ERAS implementation improved post-operative complications, LOS, and time to flatus following oesphagectomy. These results support the robust adoption of ERAS in patients indicated to undergo oesphagectomy.

## INTRODUCTION

Oesophageal cancer ranks as the sixth leading cause of global cancer mortality, with its incidence increasing six-fold in recent decades.^1–3^ Oesophagectomy, longstanding serving as the cornerstone in the curative management of oesophageal cancer, poses significant morbidity and mortality to those indicated to undergo the procedure, with rates of 59.0% and 2.4% anticipated at 30-day follow-up, respectively.^4^^,^^5^ Excessive physiological derangement following esophagectomy may negatively impact survival outcomes, attenuating the requirement for perioperative optimisation strategies in those indicated to undergo curative surgery.^6^ Accordingly, the importance of enhanced recovery after surgery (ERAS) protocols (or ‘fast track pathways’) are now recognised in the surgical management of oesophageal cancer; Initially reported by Henrik Kehlet in the 1990s, ERAS strategies aim to augment the patient experience through alleviating surgical stress, expediting postoperative recovery, and limiting post-operative complications, thus enhancing patients’ quality of life following surgery. Moreover, ERAS protocols are perceived to serve healthcare economies through facilitating shorter hospital stays and subsequently reducing healthcare associated costs and productivity.^7^ Given these reported advantages, expert consensus statements and guidelines have since been endorsed for the disciplines of colorectal,^8^ bariatric,^9^ gastric,^10^ liver,^11^ and gynaecological surgery.^12^ ERAS protocols are designed to be multifaceted such that components, such as preoperative counselling, nutritional optimisation, and early mobilisation, work synergistically to facilitate an accelerated recovery after surgery.^13^^,^^14^

The ERAS Society and Study Group published the first consensus guidelines on ERAS protocol implementation in oesophageal cancer surgery in 2019, following extensive review of the evidence surrounding individual protocol elements.^15^ Despite these recommendations, it remains unclear whether protocol implementation in oesophageal cancer surgery demonstrates a similar effectiveness to those successfully established in colorectal and other surgical specialties.^8^^,^^16^ Thus, the aim of this systematic review and meta-analysis of randomised clinical trials (RCTs) was to evaluate the impact of ERAS protocols relative to standard care (SC) in patients indicated to undergo oesphagectomy for oesophageal cancer.

## METHODS

This systematic review was conducted in accordance with the preferred reporting items for systematic reviews and meta-analyses guidelines.^17^ Ethical approval was not required from the local institutional review board due to this study using data from previously published journal articles. All authors contributed to formalising the study protocol and it was then registered with the International Prospective Register of Systematic Reviews (PROSPERO—CRD42023450309).

### Search strategy

An electronic search was performed of the PubMed, EMBASE, and Cochrane (CENTRAL) databases for relevant RCTs published between January 1990 and September 2023, which would be suitable for inclusion in this study. Included studies were limited to those published in the English language and of prospective randomised design. Studies were not restricted based on year of publication. The search was performed using predetermined keywords including; ‘enhanced recovery after surgery’, ‘fast-track protocol’, and ‘oesophageal cancer’, ‘oesophagectomy’, ‘oesophageal surgery’, and logical combinations of terms that were linked to the Boolean operator, ‘AND’. For retrieved studies, titles and abstracts were screened individually by two separate authors before full text review to deem appropriate for inclusion.

### Study selection

Eligibility criteria included RCTs with greater than 30 patients in each arm. Primary outcome measures included overall complication rate, anastomotic leak rate, and 30 day re-admission rate. Secondary outcome variables included mean length of hospital stay (LOS) (days), mean time to passage of flatus (hours), cardiac complications, pulmonary complications, gastrointestinal complications, wound complications, and patient mortality.

### Data extraction and quality assessment

The literature search was conducted by two independent reviewers (P.K and M.G.D), using a predesigned search strategy designed by the senior authors. Duplicate studies were manually removed. Each reviewer then reviewed the titles, abstracts and/or full texts of the retrieved manuscripts to ensure all inclusion criteria was met, before extracting and charting the relevant data in an Excel spreadsheet (Microsoft Corp, Redmond, WA). Data included first author name, year of publication, study design (including intervention and control), country of research facility, total number of patients indicated to undergo oesophagectomy, number of patients randomised to ERAS and SC protocols, inclusion criteria, and surgical outcome data (as outlined in the PICO framework). Risk of bias and methodological assessment of included studies was undertaken using the Risk of Bias 2.0 assessment for RCTs.

### Statistical analysis

Descriptive statistics were used to determine the associations between ERAS and SC with surgical outcomes (Fischer’s Exact Test, †).^18^ Thereafter, outcomes of patients randomised to ERAS and SC were expressed as dichotomous or continuous outcomes, reported as odds rations with their corresponding 95% Confident Intervals (CIs), following estimation using the Mantel–Haenszel method. Random effects modelling was applied to all outcome measures included in the analysis, to ensure that inter study heterogeneity was accounted for and not confounding or influencing results. Effect sizes for variables were expressed as mean difference (MD) for continuous variables and odds ratio or risk difference for dichotomous variables with 95% confidence interval. All tests of significance were two-tailed with *P* < 0.05 indicating statistical significance. Descriptive statistics were performed using the Statistical Package for Social Sciences version 26 (International Business Machines Corporation, Armonk, New York). Meta-analysis was performed using Review Manager (RevMan), Version 5.4 (Nordic Cochrane Centre, Copenhagen, Denmark). Statistical analysis was ratified by a statistician.

## RESULTS

### Literature search

**Figure 1:**
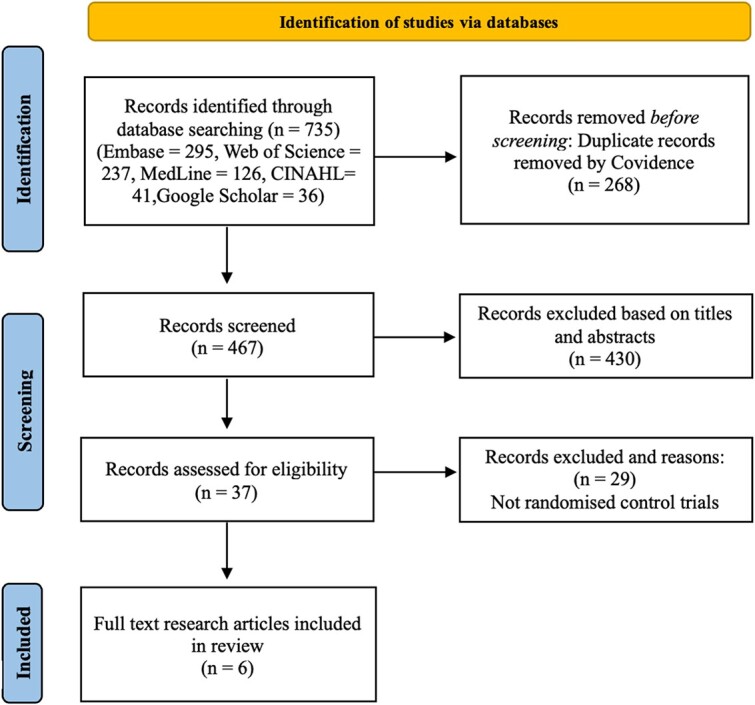
Prisma Flow Diagram

The search identified a total of 735 studies, of which 268 duplicate results were manually removed. The remaining 467 studies were screened for relevance, after which 37 had their full texts reviewed. In total, six RCTs met the eligibility criteria and were included in this study (Fig. 1).

### Study and patient characteristics

In total, 850 participants were included in this meta-analysis. Patients were enrolled between January 2008 to August 2018. In total, 424/850 (49.88%) were randomised to undergo ERAS and 426/850 (50.12%) to SC. Of these, 66.7% of patients enrolled in this study were male (567/850) compared to 33.3% females (283/850). The mean age of participants was 61.8 years old. The mean body mass index was 23.33 kg/m^2^. A detailed breakdown of study characteristics and patient characteristics of the six RCTs are depicted in Table 1 and Table 2 respectively. A detailed breakdown of the individual components making up each of the ERAS protocols from each respective RCT is outlined in Table 3.

**Table 1:** Study data from the 6 included prospective, randomised clinical trials

**Author (Year)**	**County**	**Time Period**	**Study**	**Journal**	***ERAS N***	***SC N***	**Total *N***
**Zhao (2014)**	China	Nov 2009–Mar 2011	RCT	Support Care Cancer	34	34	68
**Wang (2015)**	China	Jan 2008–Apr 2014	RCT	Asia Pac J Clinical Nutrition	90	90	180
**Chen (2016)**	China	Oct 2013–Dec 2014	RCT	BMC Cancer	128	132	260
**Li (2017)**	China	Jan 2015–Jun 2016	RCT	Journal of Thoracic Disease	55	55	110
**Zhang (2017)**	China	Jun 2012–Jun 2016	RCT	JBUON	57	57	114
**Shen (2022)**	China	Mar 2016–Aug 2018	RCT	Surgical Endoscopy	60	58	118

*RCT Randomised Control Trial, ERAS Enhanced Recovery After Surgery, SC Standard Care*

**Table 2:** Patient characteristics from the six included prospective, randomised clinical trials

**Author**	**Type of Surgery**		**Age**		**Body Mass Index (kg/m2)**		**Male:Female**	
	**ERAS**	**Standard**	**ERAS**	**Standard**	**ERAS**	**Standard**	**ERAS**	**Standard**
**Shen (2022)**	Three Stage MIE	Three Stage MIE	62.28 ± 6.74	62.33 ± 7.47	25.58 ± 2.93	23.24 ± 2.92	73.3%: 26.7%	82.8%: 17.2%
**Zhao (2014)**	One (54.6%), Two (34.5%), Three Incisions (10.9%)	One (57.9%), Two (29.8%), Three Incisions (12.3%)	55.14 ± 10.65	57.86 ± 11.34	22.52 ± 2.76	23.19 ± 2.06	79.4%: 20.6%	73.5%: 26.5%
**Wang (2015)**	Only anastomosis type reported	Only anastomosis type reported	49% >60	54% >60	N/R	N/R	67.8%: 32.2	65.6%: 34.4%
**Chen (2016)**	51.6% (MIE)^*^: 48.4% Thoracotomy	50.0% (MIE)^*^^*^: 50% Thoracotomy	56.4 ± 13.3	55.7 ± 10.3	22.53 ± 2.85	22.89 ± 2.56	80.5%: 19.5%	80.3%: 19.7%
**Li (2017)**	MIE	MIE	67.7 ± 6.7	67.0 ± 5.6	N/R	N/R	69.1%: 30.9%	74.6%: 25.4%
**Zhang (2017)**	Ivor-Lewis	Ivor-Lewis	66.89 ± 13.45	67.01 ± 12.78	N/R	N/R	31.6%: 68.4%	33.3%: 66.6%

*ERAS, enhanced recovery after surgery; MIE, minimally invasive esophagectomy*

Data reported as mean ± standard deviation ^*^Median ^*^^*^Hybrid or pure VATS

**Table 3:** Components of the enhanced recovery after surgery protocols for each randomised clinical trial included in this study

	**Shen (2022)**	**Zhao (2014)**	**Wang (2015)**	**Chen (2016)**	**Li (2017)**	**Zhang (2017)**
**Pre-operative**						
Pre-op rehabilitation training/education	+	+		+	+	+
Nutritional assessment			+		+	
Fasting strategy	+	+	+	+	+	+
Carbohydrate loading	+	+	+	+		+
Bowel cleansing					+	
**Intra-operative**						
Goal directed fluid therapy	+		+			
Blood transfusion strategy		+		+		
Maintaining normothermia		+	+	+	+	+
Pneumatic compression stockings					+	+
Pre-anaesthetic medication		No diazepam use	Dexamethasone	No diazepam use		
Anaesthesia		General + epidural anaesthesia + early extubation	General + epidural anaesthesia	General + epidural anaesthesia		Combined IV-inhalation anaesthesia (rapid metabolism + short half-life)
Nasogastric tube	−	−		−		
Nasojejunal tube	+		+			
Chest drain		+	+	+	+	+
Mediastinal drain	+					
Abdominal and cervical drain	−	−		−		
**Post-operative**						
ICU stay	−	−	+	−		−
Epidural PCA		+	+	+		
Early mobilization	+		+	+	+	
Post-op albumin administration				+		
Early catheter removal				+	+	
Chest drain on suction				+		
Removal of chest drain	<200mls		<200mls	POD3	POD1	
Jejunostomy enteral feeding		+		+	+	
Early oral intake and progression	POD5		+	+	+	+
Aspiration precautions/education				+	+	

*ERAS,* enhanced recovery after surgery; *ICU,* intensive care unit; *POD,* postoperative day

### Overall complications

**Figure 2:**
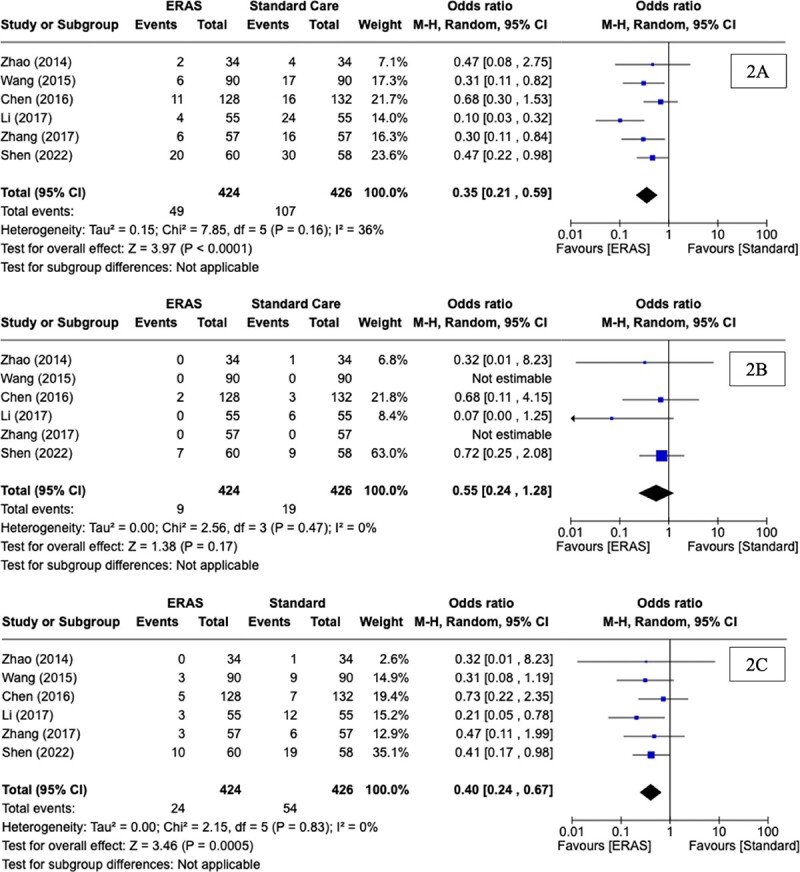
Forest plots for (A) overall complications, (B) anastomotic leak, and (C) pulmonary complications for comparison of enhanced recovery after surgery and standard care protocols following oesophagectomy

Overall complication rates were reported in all six RCTs. The overall complication rate was 18.40% (156/850). Using descriptive statistics, a significant difference was observed in the overall complication rate between those randomised to ERAS (11.56%, 49/424) versus SC (25.12%, 107/426) (*P* < 0.0001*,* †). At meta-analysis, there was a significant difference observed in complications in favour of ERAS (OR: 0.35, CI: 0.21, 0.59, *P* < 0.0001, *I^2^* = 36%) (Fig. 2A). Descriptive statistical analysis of outcomes for patients randomised to ERAS and SC after oesphagectomy are outlined in detail in Table 4.

**Table 4:** Descriptive statistical analysis of outcomes for patients randomised to ERAS and SC protocols after oesophagectomy

**Parameter**	**ERAS**	**Standard Care**	**P-Value**
**Overall complications**	11.56% (49/424)	25.12% (107/426)	<.0001
**Anastomotic leak**	2.12% (9/424)	4.46% (19/426)	0.17
**Gastrointestinal complications**	2.44% (9/369)	4.58% (17/371)	0.11
**Pulmonary complications**	5.66% (24/424)	12.68% (54/426)	0.0005
**Cardiac complications**	1.9% (7/369)	2.16% (8/371)	0.78
**30 day re-admission rates**	2.47% (4/162)	1.8% (3/166)	0.73
**Wound complications**	1.42% (6/424)	1.64% 7/426	0.78
**Mortality**	1.22% (3/245)	0.80% (2/247)	0.71

### Anastomotic leak

Anastomotic leak rates were reported in all six RCTs. The overall anastomotic leak rate across the six RCTs was 3.29% (28/850). Using descriptive statistics, there was no difference observed in anastomotic leak rates between the ERAS group (2.12%, 9/424) versus the SC group (4.46%, 19/426) (*P =* 0.082, †). At meta-analysis, there was no significant difference observed in anastomotic leak rates between those randomised to ERAS and SC (OR: 0.55, CI: 0.24, 1.28, *P* = 0.17, *I^2^* = 0%) (Fig. 2B).

### Pulmonary complications

Pulmonary complication rates were reported in all six RCTs. The rate of pulmonary complications across the six RCTs was 9.18% (78/850). Using descriptive statistics, a significant difference was observed in the rate of pulmonary complications between the ERAS group (5.66%, 24/424) versus the SC group (12.68%, 54/426) (*P* = 0.0005, †). At meta-analysis, there was a significant difference observed in pulmonary complication rates in favour of ERAS (OR: 0.40, CI: 0.24, 0.67, *P* = 0.0005, *I^2^* = 0%) (Fig. 2C).

### Cardiac complications

**Figure 3:**
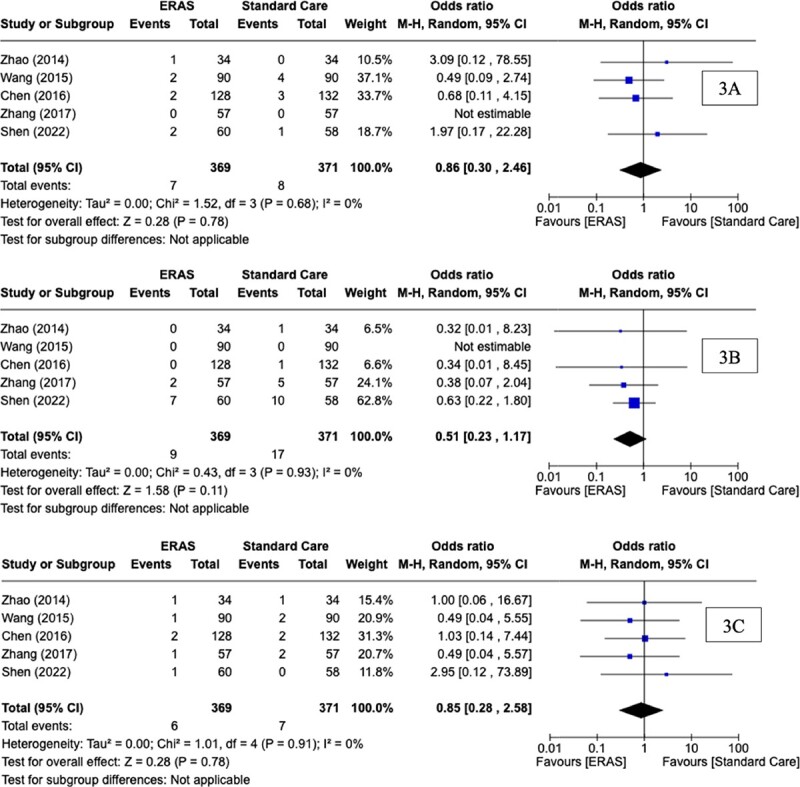
Forest plots for (A) cardiac complications, (B) gastrointestinal complications, and (C) wound complications for comparison of enhanced recovery after surgery and standard care protocols following oesophagectomy

Cardiac complication rates were reported in five of the six RCTs. The overall rate of cardiac complications across the five RCTs was 2.03% (15/740). Using descriptive statistics, there was no difference observed in cardiac complication rates between the ERAS group (1.9%, 7/369) and SC group (2.16%, 8/371) (*P* = 0.78, †). At meta-analysis, there was no significant difference observed in cardiac complication rates between groups (OR: 0.86, CI: 0.30, 2.46, *P* = 0.78, *I^2^* = 0%) (Fig. 3A).

### Gastrointestinal complications

Gastrointestinal complication rates were reported in five of the six RCTs. The overall rate of gastrointestinal complications across five RCTs was 3.5% (26/740). Using descriptive statistics, there was no difference observed in gastrointestinal complication rates between the ERAS group (2.44%, 9/369) and SC group (4.58%, 17/371) (*P* = 0.11, †). At meta-analysis, there was no significant difference observed in gastrointestinal complication rates between groups (OR: 0.51, CI: 0.23, 1.17, *P* = 0.11, *I^2^* = 0%) (Fig. 3B).

### Wound complications

Wound complication rates were reported in five of the six RCTs. The rate of wound complications across five RCTs was 1.76% (13/740). Using descriptive statistics, there was no difference observed in wound complication rates between the ERAS group (1.42%, 6/369) and SC group (1.64%, 7/371) (*P* = 0.78, †). At meta-analysis, there was no significant difference observed in wound complication rates between groups (OR: 0.85, CI: 0.28, 2.58, *P* = 0.78, *I^2^* = 0%) (Fig. 3C).

### Thirty day readmission rate

**Figure 4:**
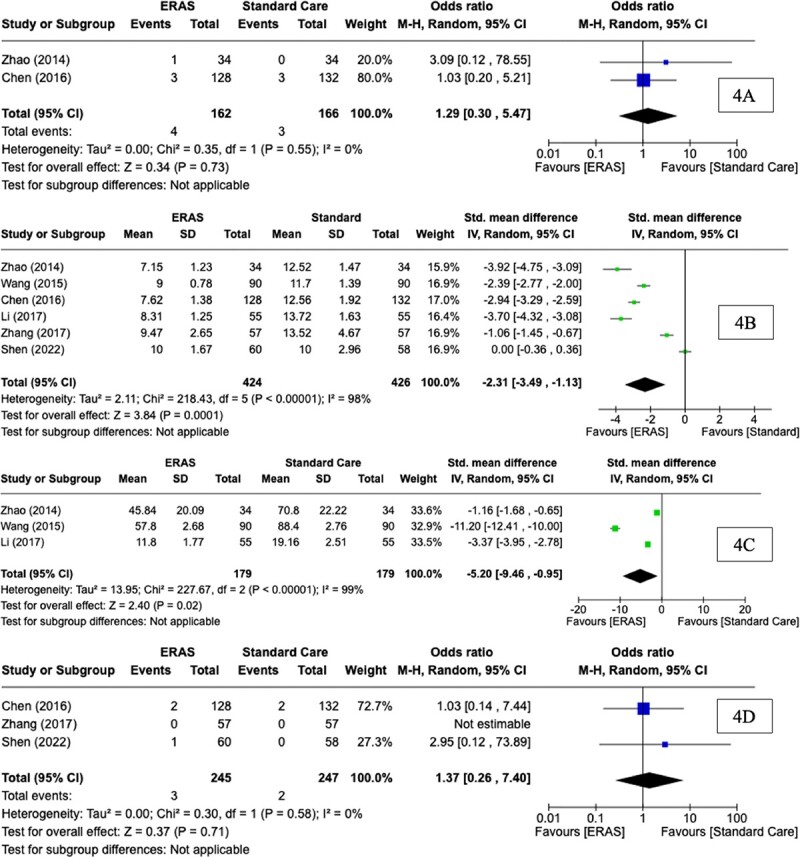
Forest plots for (A) 30 day re-admission, (B) post-operative length of stay, (C) post operative flatus time, and (D) mortality for comparison of enhanced recovery after surgery and standard care protocols following oesophagectomy

Thirty day readmission rates were reported in two of the six RCTs. The 30 day re-admission rate of two RCTs was 2.13% (7/328). Using descriptive statistics, there was no difference observed in thirty day re-admission rates between the ERAS group (2.47%, 4/162) and SC group (1.8%, 3/166) (*P* = 0.73, †). At meta-analysis, there was no significant difference observed in thirty day re-admission rates between groups (OR: 1.29, CI: 0.30, 5.47, *P* = 0.73, *I^2^* = 0%) (Fig. 4A).

### Post-operative length of stay

Post-operative LOS was reported in all six RCTs. Using descriptive statistics, a significant difference was observed in the mean LOS between the ERAS group (Mean: 8.59 days) and SC group (Mean: 12.34 days) (*P* < 0.0001, †). At meta-analysis, there was a significant difference observed in the mean LOS between groups (MD: -2.31, CI: −3.49, −1.13, *P* < 0.0001, *I^2^* = 98%) (Fig. 4B).

### Post-operative time to flatus

Post operative flatus time was reported in three of the six RCTs. Using descriptive statistics, a significant difference was observed in the post-operative time to flatus between the ERAS group (mean: 38.48 hours) and SC group (mean: 59.45 hours) (*P* = 0.020, †). At meta-analysis, there was a significant difference observed in the post-operative time to flatus between groups (MD: -5.20, CI: −9.46, −0.95, *P* = 0.02, *I^2^* = 99%) (Fig. 4C).

### Mortality

Mortality rate was reported in three of the six RCTs. The total mortality rate of three RCTs was 1.06% (5/492). Using descriptive statistics, no significant difference was observed in mortality rates between the ERAS group (1.22%, 3/245) and SC group (0.80%, 2/247) (*P* = 0.710, †). At meta-analysis, there was no significant difference observed in mortality rates between groups (OR: 1.37, CI: 0.26, 7.4, *P* = 0.71, *I^2^* = 0%) (Fig. 4D).

## DISCUSSION

This systematic review and meta-analysis incorporated data from six prospective randomised trials comparing post-operative outcomes following ERAS protocol implementation following oesphagectomy. Results from this rudimentary meta-analysis demonstrate that favourable outcomes may be anticipated for those who adhere to ERAS protocols, in particular, reduced overall complication rate, pulmonary complications, LOS, and post-operative time to flatus. While this study demonstrated no significant difference in the remaining outcome measures including complications (including anastomotic leak, wound, cardiac, and gastrointestinal), 30-day re-admission rates, and mortality, it is important to note some clinically significant findings, for example, a greater than 100% relative increase in the risk of anastomotic leak rates in the standard care recipients, albeit falling shy of statistical significance. Furthermore, the overall complication rate was two-fold higher in the SC group (25.12%) relative to the ERAS group (11.56%). Thus, with this in consideration, in tandem with the aforementioned significant findings, the authors can fairly attest that ERAS robust implementation should be considered where feasible, due to it being clinical practical in improving post-operative surgical outcomes in the setting of oesphagectomy.

While this study highlights an obvious benefit associated with the use of ERAS protocols post oesphagectomy, it is important to highlight the considerable variability within each of the protocols across the six included RCTs. In conglomeration, these protocols evidently improve post-operative outcomes for these patients, stemming from the theoretical pragmatism in attempting to subdue potentially detrimental effects posed by the physiological and immunogenic cascade associated with the insult of oesphagectomy on both the local and systemic tissues. Nevertheless, there is a lack of apparent consensus with what should be integrated into the esophagectomy ERAS protocol, which is likely due these templates being designed for use for oncological resections for other surgical specialties (i.e. colorectal and cardiothoracic surgery), then subsequently improvised to encompass resections of the early gastrointestinal tract. Therefore, the authors suggest that future efforts be focused upon developing ERAS protocols specifically tailored to address the distinct challenges associated with oesophageal resection, as opposed to being adapted from other complex major procedures. Notwithstanding, it is important to note the efforts of the ERAS Society and Study group: Their 2018 publication was designed with the aim to inform protocols specifically for oesphagectomy using the best available evidence,^15^ via highlighting how a multifaceted, multimodal approach to ERAS implementation following oesphagectomy which includes each of the stakeholders responsible for delivering perioperative care to these patients. While it is challenging to identify the critical elements responsible for the beneficial effects seen in the ERAS groups across the six RCTs, the ERAS Society and Study Group have documented a high level of evidence to support the implementation of specific protocol elements in the peri-operative care of oesophageal cancer surgery. The highest level of evidence in these recommendations relate to the avoidance of hypothermia, consideration of expeditious removal of urinary catheters, avoidance of pre-operative prolonged fasting, use of appropriately dosed intermediate acting muscle relaxants, and optimisation of fluid balance.^15^ While not applied universally across the six included studies, all elements are represented in some respect across protocols. While these authors do explicitly provide recommendations to what should be included in such protocols, the impact of these recommendations will likely be seen in future studies with a reduction in protocol variability.

This study is subject to unavoidable limitations. Primarily, while the variability in the ERAS protocols will inevitably limit the robustness and direct transferability of these results into the clinical setting. Secondly, as with all RCTs in surgery, the apparent inability to blind surgeons and other stakeholders in patient care to the intervention inevitably lends such studies to be classified as ‘open label’ rendering them subject to potential biases.^19^ A further limitation to this meta-analysis is that only two studies reported mortality rate and three studies reported thirty day re-admission rates. These outcome measures warrant inclusion as they represent key markers of ERAS protocol effectiveness. Finally, all the included trial in this study were from Chinese research institutes; the transferability of these results to patients in other parts of the world may be challenging, in particular when varying genetic, cultural and surgical practices vary considerably worldwide.

In conclusion, this study demonstrates favourable outcomes in respect to overall complications, pulmonary complications, LOS, and post-operative time to flatus in favour of patients randomised to ERAS protocols following oesphagectomy, thus demonstrating the safety of robust implementation where feasible. Accordingly, it is apparent that implementation of ERAS programs should be considered for patients indicated to undergo resection for oesophageal carcinoma.

## Sources of funding

None received.

## ABBREVIATIONS

BMI, body mass index; CI, confidence interval; ERAS, enhanced recovery after surgery; ICU, intensive care unit; LOS, length of stay; MD, mean difference; MIE, minimally invasive esophagectomy; OR, odds ratio; POD, postoperative day; PRISMA, preferred reporting items for systematic reviews and meta-analyses; RCT, randomised control trial; SC, standard care; SPSS, statistical package for social sciences

## SPECIFIC AUTHOR CONTRIBUTIONS

Patrick Kennelly (Formal analysis, Investigation, Methodology, Software, Writing—original draft, Writing—review & editing), Noel Donlon (Resources, Supervision, Validation, Writing—review & editing), Matthew G. Davey (CRediT contribution not specified), Diana Griniouk (Resources, Writing—original draft), Gavin Calpin (Formal analysis).

## Data Availability

Data will be made available upon reasonable request from the corresponding author. International Prospective Register of Systematic Reviews (PROSPERO—CRD42023450309).
